# Assessment of Surface Geometry of Puckered Area of SeerSucker Woven Fabrics

**DOI:** 10.3390/ma19173648

**Published:** 2026-08-27

**Authors:** Malgorzata Matusiak

**Affiliations:** Lodz University of Technology, Faculty of Textiles and Design, Institute of Architecture of Textiles, Zeromskiego 116 str., 90-543 Lodz, Poland; malgorzata.matusiak@p.lodz.pl

**Keywords:** seersucker woven fabrics, surface, roughness, waviness, profilometer, measurement

## Abstract

The crucial feature of seersucker woven fabrics is their surface geometry. In such kinds of woven fabrics, the puckered stripes appear in the warp direction, separated by stripes of smooth fabric. There are practically no methods to assess the seersucker effect and the reproducibility of this effect. In this work, an optical profilometer enabling non–contact assessment of surface geometry was used to measure the shape of the puckered stripes of example seersucker woven fabrics. Waviness amplitude parameters, such as the arithmetic mean height of individual points within the evaluation area, root mean square deviation of points’ height within the evaluated area, maximum waviness height, maximum peak height, maximum valley depth, skewness and kurtosis, were analyzed for the puckered stripes of the fabrics. Profiles along the puckered stripes were created to analyze their shape. The results showed that weft yarn type influences the waviness amplitude parameters and shape of the puckered stripes in a statistically significant way. To summarize the results, weft yarns with higher linear density provide the most intense seersucker effect, meaning a wave with the greatest peak height and greatest trough depth. Conversely, weft yarns with the lowest linear density provide less variation in peak height and valley depth but the highest wave frequency. However, the observed differences are associated with the weft yarn type, with linear density being only one of several possible explanatory factors.

## 1. Introduction

**Figure 1:**
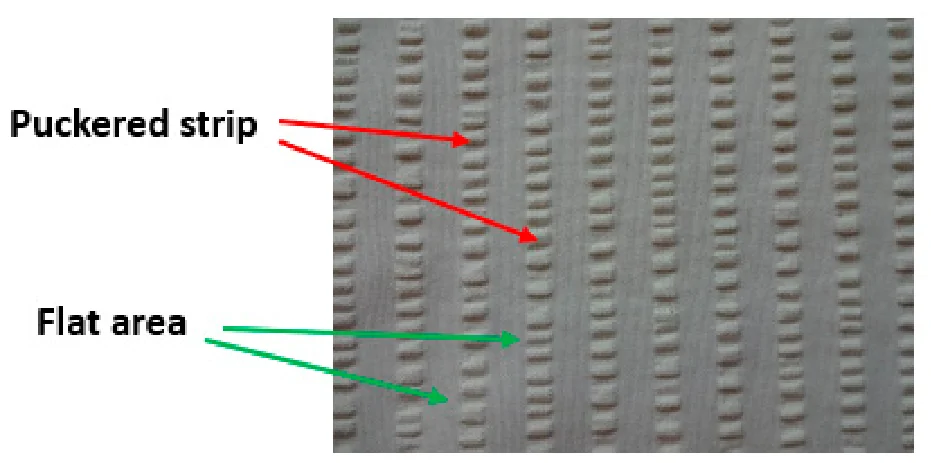
Example picture of the seersucker woven fabric.

Seersucker woven fabrics are a specific kind of woven fabric of unique structure and properties. They are characterized by the fact that two phases can be distinguished on the fabric surface: a convex––concave phase and a smooth/flat phase [1,2,3]. The convex––concave area is created by the stripes running in the warp direction, separated from each other by a smooth phase of fabric (Figure 1). The width of these puckered stripes can be uniform across the entire width of the fabric, or it can vary. The distance between these stripes can also be the same or different. This depends on the fabric designer’s concept. Technological aspects, i.e., the conditions on the loom on which the fabric is produced, also play an important role in shaping the surface topography of these fabrics.

Seersucker fabrics can be manufactured using different methods. Generally, there are three main methods listed in the literature [4,5]:

- Finishing,
- Thermal,
- Weaving.

In the finishing method, the pattern on the fabric surface is achieved by using specially shaped rollers under appropriate conditions. The process involves chemical and thermal action. Next, the effect is fixed. In the thermal method, the seersucker effect is created thanks to the application of yarns with varying degrees of shrinkage under temperature. In the thermal method, the seersucker effect can be achieved in both the warp and weft directions. The method can also be applied in shaping the seersucker effect onto knitted fabric surfaces. In the finishing method, chemical and thermal treatment causes a reduction in fabric strength. However, the thermal method cannot produce fabrics with a uniform raw material composition, nor can it produce fabrics made from natural fibers. These limitations do not apply to the weaving method.

This is a mechanical method in which the seersucker effect is achieved directly on the loom during the weaving process. This method allows for different ways of achieving zonal variation in fabric structure:

- Application of different numbers of warp threads in the zones forming the base and puckered areas of the fabric,
- Application of different linear densities of both the base and puckering warp threads,
- Varying the tension of warp threads during fabric manufacturing,
- Application of different weaves in the base and puckered fabric zones,
- Mixing all the above–mentioned ways.

The weaving method is the most common method of manufacturing seersucker woven fabric. The fabrics created using this method are characterized by a durable seersucker effect and better mechanical properties than those manufactured using other methods: thermal and finishing. The disadvantage of seersucker woven fabrics produced by the weaving method is their greater mass per square meter and stiffness, as well as lower drapeability, compared to fabrics produced by other methods.

Seersucker fabrics are typically used in clothing, especially summer clothing, and home textiles, such as bed linen. They have been known and used for years, but publications on their production, especially topography shaping, and their properties are relatively few. One of the reasons for this may be the fact that standard procedures described in the relevant standards cannot always be used to measure the properties of seersucker woven fabrics [6].

Due to their design and functional properties, seersucker woven fabrics are widely used in clothing. However, their production is limited. The reason for this, especially in the case of fabrics produced by the weaving method, is that the loom efficiency for their production is lower than that achieved with standard flat fabrics’ manufacturing. This is due to the high density of the fabrics, especially in the area of the puckered stripes. At the same time, yarn consumption for seersucker fabrics is higher than that for flat fabrics with standard weaves. This is the result of very high take–up (50% or more) of the warp forming the puckered stripes.

Another limitation in the production of seersucker fabrics using the weaving method is implementing different tensions on both warp sets; creating flat and puckered areas on the fabric is a difficulty in ensuring the seersucker (puckered) effect is repeatable. A number of factors related to loom settings and yarn parameters mean that subsequent batches of fabrics may differ in terms of both the durability of the seersucker effect and the shape of the puckered stripes. Even using yarns of the same linear density in the warp and weft does not ensure reproduction of the geometry of the puckered stripes from the previous batch, let us call it the reference batch. In the case of cotton yarns, it is assumed that the deviation of the actual linear density from the nominal can be from −5% to +3%. For example, for a yarn with a linear density of 40 tex, the range of actual linear density can be from 38.0 tex to 41.2 tex. Yarn twist can also vary. Furthermore, in the case of cotton yarns, the properties of the fibers used in subsequent batches may differ, particularly their maturity, which affects fiber elasticity. This means that after the seersucker fabric is removed from the loom, the warp relaxation process, especially within the puckered stripes, may vary between different batches. This creates the possibility of achieving different seersucker effects in different batches of fabric with a theoretically identical structure.

Another problem is the lack of a method to quantify the seersucker effect, and, thus, to objectively check the repeatability of the shape of puckered stripes in subsequent fabric batches. The only indicators that can currently be used to characterize the seersucker effect are the take–up of the warp yarns that create the puckered stripes and the relationship between the take–up of warps creating both the puckered and flat areas of seersucker woven fabrics. In standard flat fabrics of basic and derivative weaves, the warp and weft take–up is on the order of a few percent. In seersucker fabrics, the take–up of the warp creating the puckered stripes is 50% or more.

Currently, the quantification of the shape of puckered stripes, which are characteristic structural elements of seersucker fabrics, is a problem. Among other things, this is due to limited possibilities to measure the surface geometry of these fabrics. The KES–FB4 instrument is typically used to measure the surface roughness of textile materials. This is a module of the KES (Kawabata Evaluation System) designed for the instrumental assessment of fabric handle [7,8,9].

Testing the surface properties of textile materials using the KES–FB4 is a contact measurement [10]. This means that the sensor is in contact with the surface being measured. During measurement with the KES–FB4, the sensor moves relative to the surface of the puckered stripes, resulting in their deformation. Observations during the test performance show that the readings obtained from the KES–FB4 when measuring along the puckered stripes do not reflect the actual shape of the puckered stripes, but rather the instantaneous shape resulting from the deformation of these stripes due to pressure applied to the surface of the moving sensor.

Measurement of seersucker fabric surfaces using non–contact methods does not cause deformation of the puckered surfaces of these fabrics. However, testing of textile materials using non–contact methods is not widespread. Most textile laboratories do not have the equipment necessary for this type of testing. Furthermore, testing the surface structure of textile materials using non–contact methods, primarily optical ones, is not standardized. This also prevents accredited laboratories from using this measurement method.

Optical methods provide many standardized parameters characterizing the geometric structure of a surface. However, it has not yet been determined which of these parameters, or set of parameters, would allow for a numerical description of the shape of the puckered strips in seersucker woven fabrics. All parameters characterize the surface geometry. However, the proper selection of parameters or functions should be made for the particular purpose of measurement and analysis. In the literature published to date, recommendations regarding this aspect have not been made. 

The measurement of surface geometry by means of non–contact 3D methods is becoming increasingly common in the measurement of solid objects such as machine components or ceramic materials. Parameters characterizing the geometric structure of surfaces obtained from non–contact methods are defined in relevant international standards. Non–contact methods are also used to measure the surface geometry of textile materials. However, research in this area is relatively sparse. Militky et al. [11] applied an image analysis to obtain images of fabric surface profiles. Modrak [12] investigated the topography of fibrous materials by means of TALYSURF CLI–500—a high–resolution contactless 3D surface profiling system. Mohri et al. [13] assessed a fabric wrinkle by using the image analysis technique utilizing the radon transform (RT) and texture analysis. Calvimontes et al. [14,15] investigated the topography of textile materials using a length scale concept in order to characterize surface geometry by analyzing their specific morphologies resulting from a type of weave, a kind of yarn, and fibers. They reported the correlation of topographical changes caused by different modification processes. Korzeniewska et al. [16] compared two methods for measuring parameters characterizing the surface topography of coated fabrics: a contact method using a profilograph and a contactless method using an optical sensor (OCT). The experimental results confirm the usefulness of the optical method for assessing the surface parameters of layers applied on flexible substrates, as an alternative to contact measurements. Matusiak and Frącczak applied 3D laser scanning to evaluate the surface geometry of seersucker woven fabrics [17]. This article represents the first published study of the topography of seersucker fabrics. In this study, an experimental setup with a 3D laser scanner was applied to scan the seersucker woven fabric surface. Next, the scans were processed in the SolidEdge ST7 software. The lines created in both directions, warp and weft, were analyzed manually. A single parameter, Wz, the–maximum waviness height of a surface, was selected, based on which individual profiles were analyzed. This was the first approach to the topic, which allowed for preliminary exploration of the issue and provided the impetus for further investigations. In recent years, Matusiak and Kosiuk investigated the surface geometry of woven fabrics before and after the Martindale test by means of an optical profilometer [18]. They stated that measurements of the geometric structure of fabric surfaces using a non–contact optical method allow for an evaluation of changes in the structure of the surface subjected to the abrasion process using the Martindale device. Matusiak [19] started investigating the surface geometry of seersucker woven fabrics by means of the optical profilometer. She compared the surface geometry characteristics of flat and puckered areas of the example cotton seersucker woven fabric. Matusiak also pointed out the need for further in–depth research on the surface geometry of seersucker fabrics with different structures, especially analysis of the geometry of the puckered area of such kind of woven fabrics. The paper presented a method—the non–contact optical method—based on the measurement results of one example seersucker fabric. The author showed the parameters that can be determined using the optical method and graphs that can be provided by the software cooperating with the profilometer. Additionally, the article showed the influence of the value of cut–off filter on the obtained results, especially the shape of the fabric profiles, and proposed a cut–off filter value of 1.4 mm as more suitable for testing a given fabric than a filter of 0.8 mm, commonly used in testing the surfaces of solid materials [19].

The aim of the presented work was to measure the surface geometry of the puckered stripes of seersucker woven fabrics. It was assumed that the key to assessing the seersucker effect is to examine the puckered stripes rather than the entire seersucker stripes’ repeat. The flat phase represents a typical two–dimensional fabric with a basic weave. It creates the space between the puckered stripes and cannot be considered a characteristic of seersucker woven fabric.

Additionally, an analysis of the influence of the kind of weft yarn on the surface characteristics of the puckered stripes was performed. The weft yarns differed not only in terms of linear density but also in their raw material composition. However, data on the raw material composition were insufficient to analyze the influence of this factor on the formation of the geometry of the puckered stripes. Such an influence certainly exists, as fiber elasticity influences the fabric’s relaxation process after removal from the loom. Analyzing the effect of the weft yarn composition on the topography of the seersucker woven fabric would require initiating the experiment by producing an experimental batch of yarns with a programmably variable raw material composition. In this experiment, commercially available yarns were used to produce the fabrics.

## 2. Materials and Methods

In the present work, four variants of seersucker woven fabrics were the objects of investigation. They were fabrics manufactured on the basis of the same yarn: 20 tex × 2 cotton applied in the warp creating the puckered stripes (warp II) and the warp creating the flat area (warp I) of the fabrics. Four kinds of yarns were introduced in the weft direction:

- 20 tex × 2 cotton,
- 15.6 tex × 2 ThermoCool Fresh,
- 15 tex × 2 cotton 50%/polyester 50%,
- 12.0 tex × 2 DryRelease SeeCell active.

The ThermoCool Fresh yarn is composed of Coolmax and Thermolite fibers. Coolmax is a profiled fiber that quickly wicks moisture away from the skin and onto the material’s surface. Thermolite provides excellent thermal insulating properties due to its hollow structure. By mixing two kinds of fibers, the ThermoCool yarn combines the properties of both components. Additionally, the ThermoCool Fresh version of the yarn contains silver particles, giving an antibacterial effect. 

DryRelease is a blend of specific hydrophobic synthetic fibers with hydrophilic fibers of an optimized share of components. In the manufactured seersucker fabric, a unique version of the DryRelease yarn was applied in which the hydrophobic fibers were blended with SeeCell active fibers with antimicrobial and skin care features. SeaCell fibers combine cellulose with seaweed.

The fabrics were manufactured on the same loom, at different weft densities. Adjustment of weft density was necessary to obtain a stable and durable seersucker effect with different weft yarns. The basic structural properties of the manufactured seersucker woven fabrics are presented in Table 1. The determination of the structural parameters of the investigated seersucker woven fabrics was done using the measurement procedures standardized in the following standards:

- Mass per unit area—ISO 3801:1977 [20];
- Thread density—ISO 7211–2:1984; [21];
- Thread take–up—PN–88/P–04636 [22];
- Thickness—ISO 5084:1996 [23].

Due to the application of weft yarns of different linear densities and raw material composition, some parameters are different for particular variants. First of all, the weft density (number of picks) is different. The weft density is adjusted on the loom during the seersucker fabrics’ manufacturing. It is adjusted in such a way as to receive an appropriately strong and durable seersucker effect across the entire width of the puckered stripes. The raw material composition also plays an important role in shaping the final properties of the fabrics. After fabric removal from the loom, tension relaxation occurs in the yarns of both systems. Due to the different properties of the fibers used in the weft yarns, tension relaxation in these yarns proceeds differently, which translates into different structural properties of the fabrics, such as mass per square meter, take–up of both warp and weft yarns and thickness (Table 1). 

As previously mentioned, the only parameter that can be used to evaluate the seersucker effect is the take–up of the warp creating the puckered stripes. This is typically greater than 50%. The take–up informs us about how the warp length was introduced into the unit of fabric length. More precisely, this parameter expresses the amount of extra warp thread consumed as it curves over and under the weft threads. In the investigated seersucker woven fabrics, the take–up of the warp creating the puckered stripes is in the range from 54.5% to 70.6%. The highest warp take–up (70.6%) is found for variant No. 1—the seersucker fabric with the 20 tex × 2 weft yarn. This results from the fact that the circumference of the 20 tex × 2 weft yarn is the greatest among all weft yarns applied in the investigated fabrics. In the case of Variants No. 1, No. 2 and No. 3, no simple relation can be observed between the linear density of the weft yarns applied and the take–up of the warp creating the puckered stripes. In Variant No. 4, the thinnest weft yarn (12.0 tex × 2) was introduced but the take–up of the puckering warp (62.9%) is greater than that for Variants No. 2 (60.0%) and No. 3 (54.5%) with thicker weft yarns (Table 1). Probably, this is due to the effect of the interaction between the tension of yarns in Variant No. 4 during its manufacturing and the relaxation properties of the weft yarn. 

**Figure 2:**
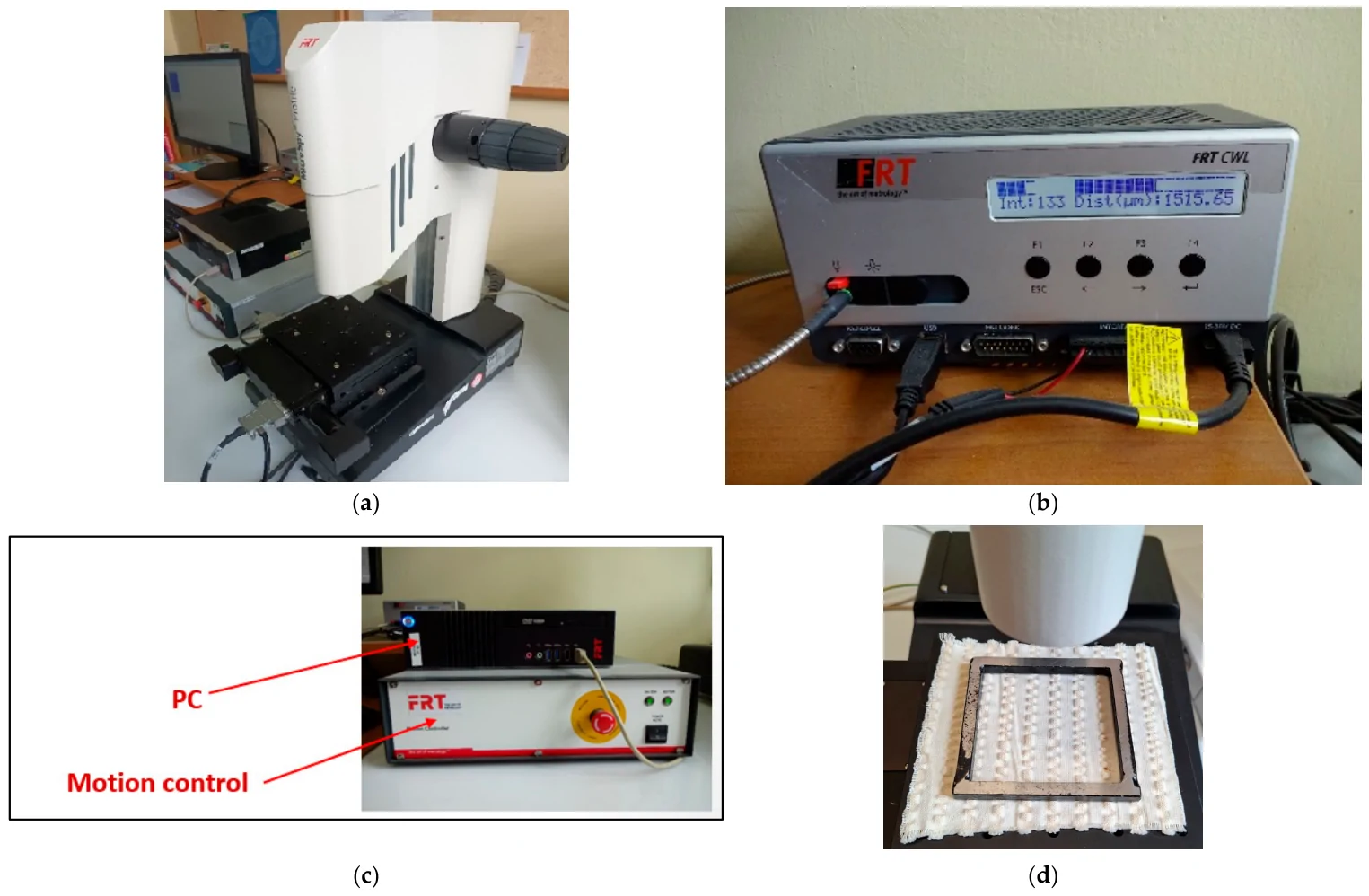
The measuring station: (**a**) optical profilometer, (**b**) CWL sensor, (**c**) motion control and PC, and (**d**) sample placement on the profilometer table.

The take–up of the puckering warp can be a measure of the intensity of the seersucker effect. However, this parameter does not provide information about the shape of the puckered stripes. For this purpose, investigations of the surface geometry of seersucker fabrics, especially the surface geometry of the puckered stripes, can be useful. Taking this into account, the MicroSpy® Profile profilometer (FRT the art of metrology™, Bergisch Gladbach, Germany) was applied to assess and analyze the surface geometry/topography of the puckered stripes in the investigated seersucker woven fabrics. The instrument belongs to the non–contact 3D optical methods of surface measurement [18,19]. The measurement station used in the research consists of the following elements: an optical profilometer (Figure 2a), motion control (Figure 2c), CWL sensor (Figure 2b) and PC (Figure 2c) (FRT the art of metrology™, Bergisch Gladbach, Germany).

The MicroSpy® Profile is a scanning measuring instrument, i.e., a device which uses an optical sensor to investigate a selective distance value. The sample is moved under program—control back and forth under the sensor, with each line having a displacement. The FRT CWL sensor used in the MicroSpy® Profile utilizes the chromatic aberration of optical lenses [24]. In the current work, the default settings of the sensor are applied: detection rate—2000 Hz, Z–range—3mm, step—2100 μm. The tests are performed on a square–shaped sample area measuring 49 mm on one side. This is the maximum surface area that can be examined using the profilometer used. The sample is placed on a measuring table, with precise computer–controlled movement. The sample is positioned so that the puckered stripes run vertically, and additionally, a central puckered strip runs through the center of the table. To ensure good adhesion of the sample to the instrument’s table surface, the sample is loaded with a metal frame (Figure 2d), specially made for these measurements. 

In the presented work, 3 repetitions of the measurement are performed (3 specimens of each seersucker fabric variant). 

**Figure 3:**
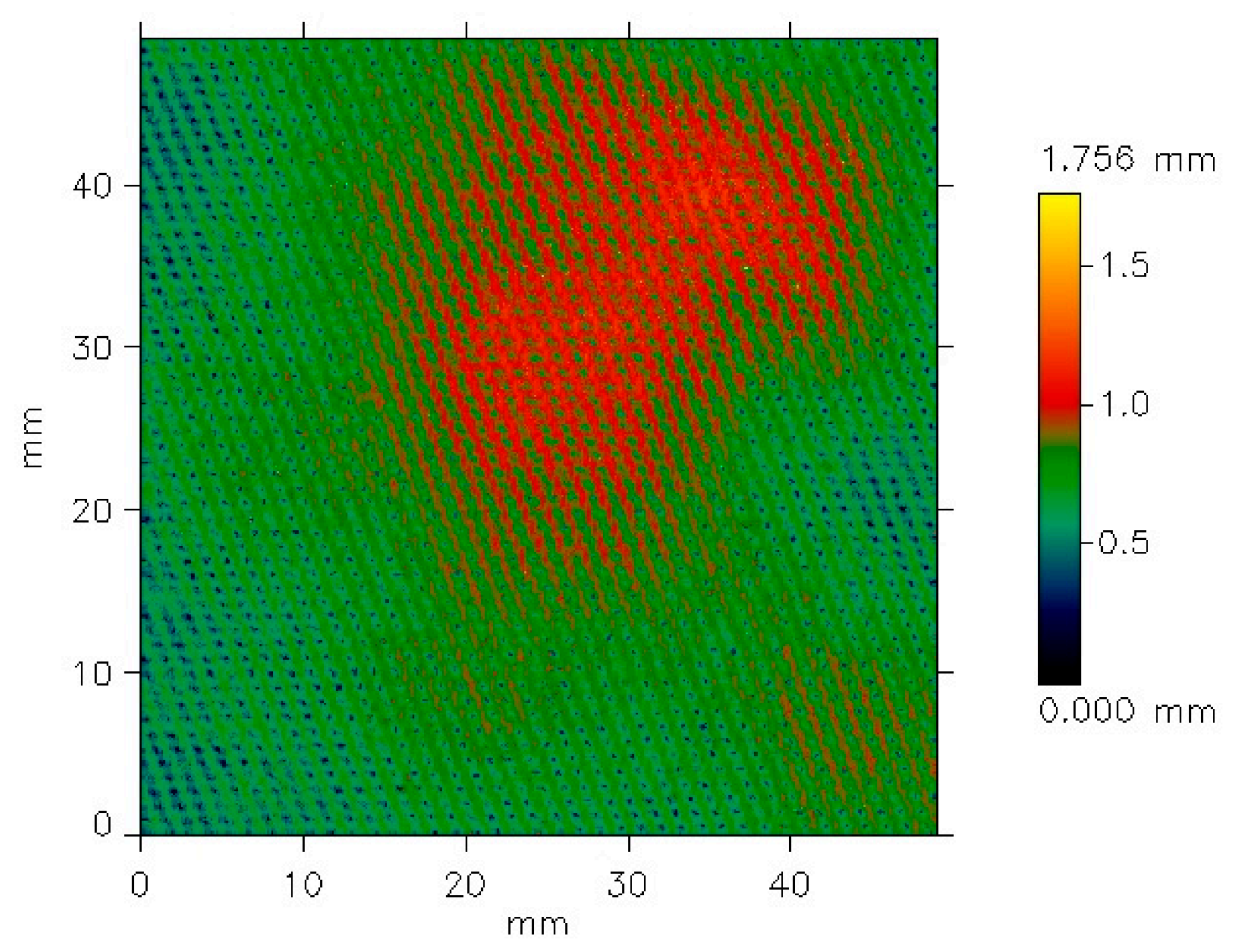
Example fabric picture from the MicroSpy® Profile profilometer.

The profilometer provides a color picture of the measured surface. In the surface images, the color corresponds to the height of individual points on the examined surface (Figure 3). 

The data from the profilometer are processed in the Mark III software version 3.11 co–operating with the instrument [25]. First, the picture needs modification in order to correct the invalid data. In the present work, areal interpolation is applied for this. 

On the basis of the height of individual points on the analyzed surface, the software calculates parameters characterizing the surface geometry according to appropriate standards [26,27]. It also provides a range of functions (autocorrelation function, power spectral density) and charts (height histogram, angle distribution, hone angle) useful in assessing the surface topography of measured objects.

In the current work, the measurement was done for a square surface with a side of 49 mm. In order to assess the seersucker effect, only the puckered stripes were analyzed because they are the crucial elements of seersucker fabrics. The flat areas are typical of fabrics of plain weave. They create the distance between the puckered stripes. In order to analyze the surface geometry of the puckered stripes, the Modification function was applied to separate the area of individual puckered stripes. The stripes were separated using the masking function and rectangle tool in the software menu. Analysis of the surface geometry parameters for the puckered stripes of the tested seersucker woven fabrics allowed for an assessment of the influence of weft yarn kind on the shape of puckered stripes. 

Generally, for all objects, three kinds of surface irregularities are distinguished: roughness, waviness and errors of form [28]. Standard textile materials are flexible and their form is variable. Due to this fact, only two other kinds of surface irregularities, roughness and waviness, are under consideration. The surface roughness consists of a set of surface irregularities that may or may not repeat periodically and are characterized by the fact that the ratio of the average width of the elements to their average height is less than 40. Surface waviness is defined as a set of periodically repeating elements at longer intervals than those of the roughness [28,29].

For the area of the puckered stripes, the parameters characterizing the waviness were determined according to standard [26]. The waviness parameters were determined after elimination of the roughness component. For this purpose, the cut–off filter λc = 1.2 mm was applied. The value of the cut–off filter was established after analyzing the smooth and puckered stripes’ profiles at various filter values. This was done by applying the procedure described in a previous publication [19]. For the tested fabrics, the λc = 1.2 mm filter allowed us to eliminate short–term surface irregularities caused by the weave (the transition of warp and weft threads from one side of the fabric to the other) from the waviness profile. Additionally, the default value of λs = 245 μm for the noise filter was applied to eliminate noise.

For each fabric variant, the analysis was done for three puckered stripes on the scanned fabric area. Next, the average values of the parameters were calculated. In order to assess the differences between the waviness parameters for particular fabric variants, one–way ANOVA, available in TIBCO Statistica software version 13.3, was applied. In the statistical analysis, a fabric variant was taken as the independent variable, whereas the waviness parameters were dependent variables. Statistical significance was assessed at a 0.05 significance level. Additionally, the post hoc–Tukey’s test and correlation analysis were performed. 

## 3. Results and Discussion

**Figure 4:**
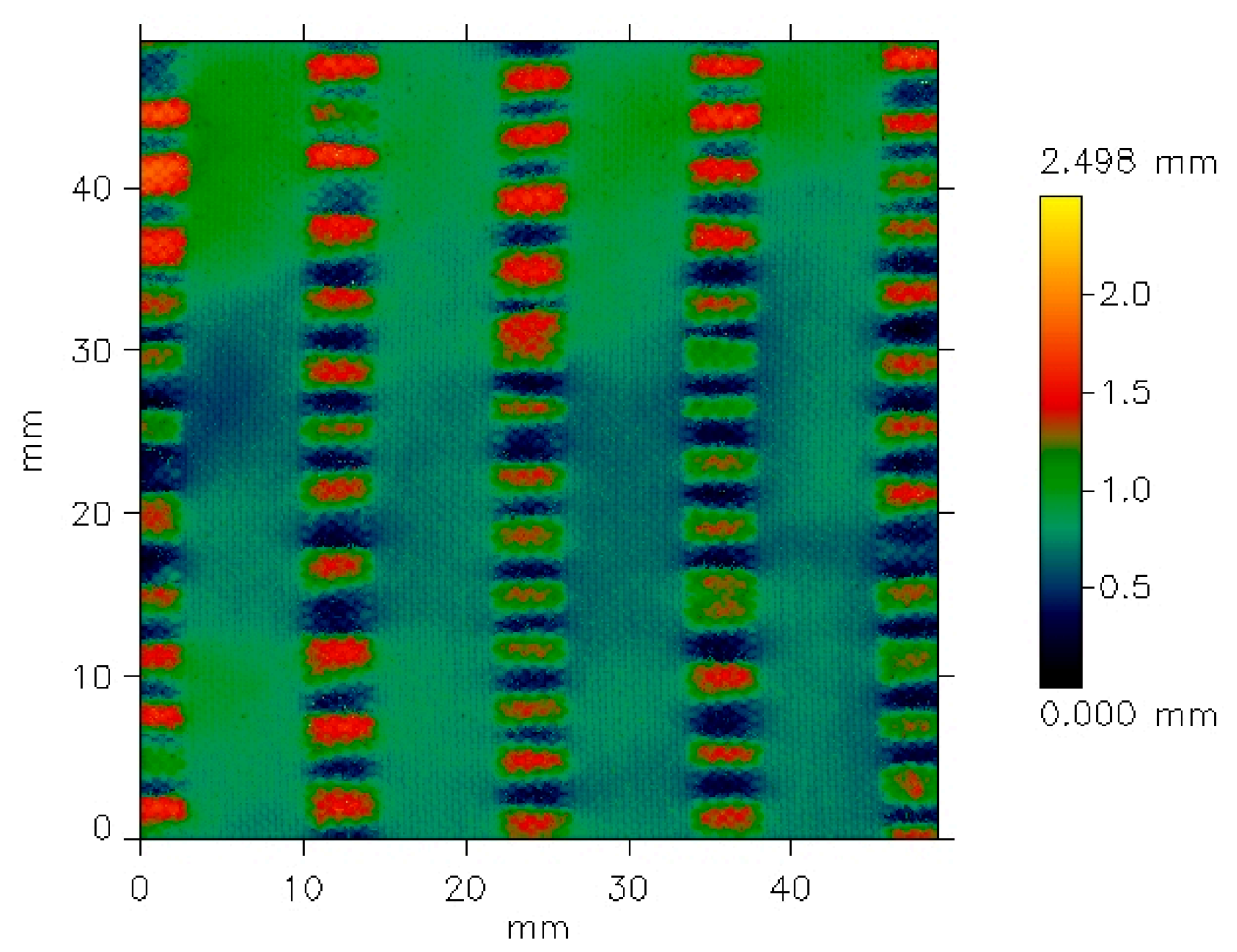
Example picture of seersucker woven fabric—Variant No. 2.

Figure 4 presents an example of a surface picture provided by the profilometer. On the right side of the picture, the scale bar is presented, in which each color represents the appropriate height of points on the measured area. In the picture, the flat and puckered areas of the seersucker woven fabric are clearly visible and distinguished. 

**Figure 5:**
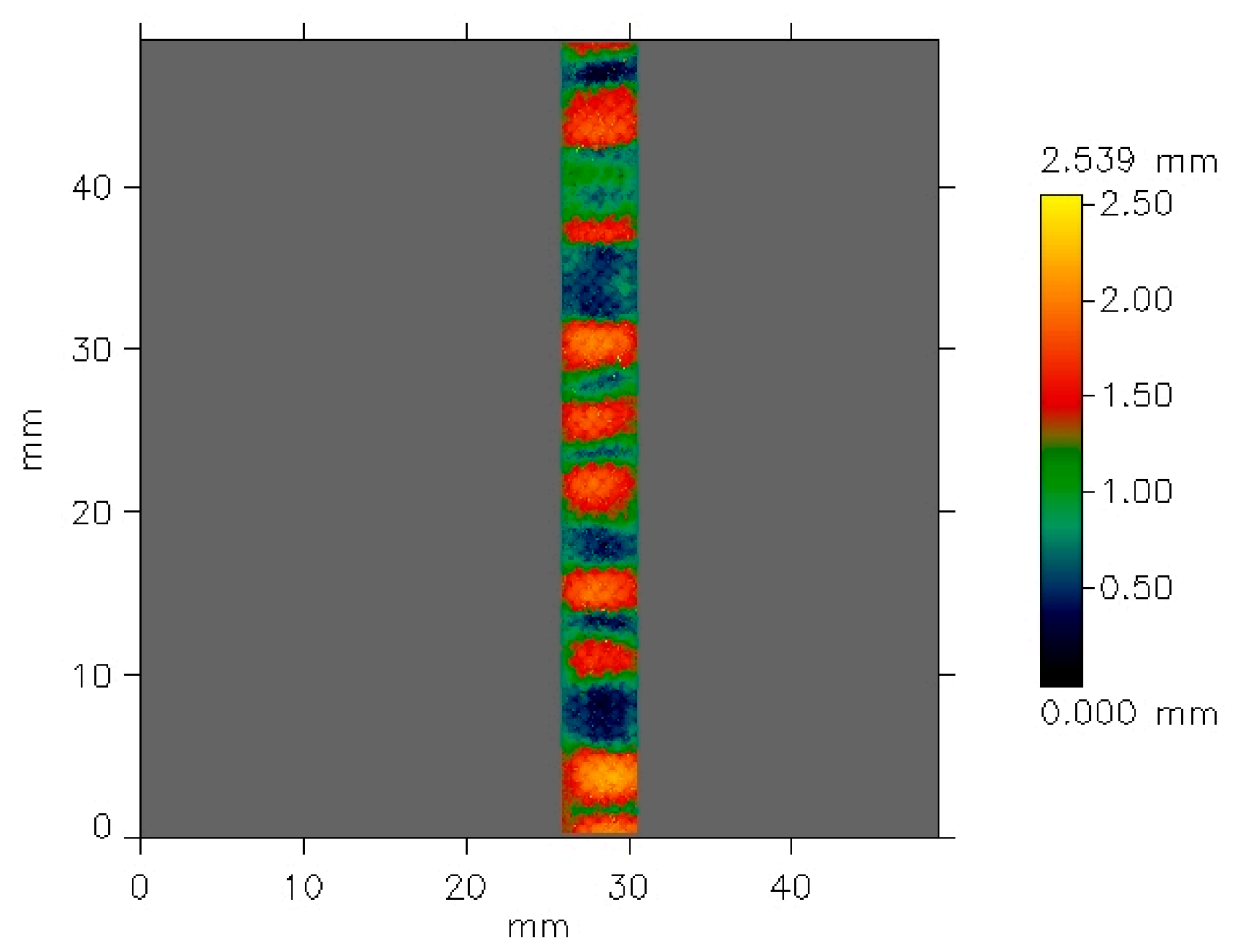
Example area of puckered strip of Variant No. 1.

From the entire area of the fabric picture, three puckered stripes were separated. The picture below (Figure 5) presents an example area of a puckered stripe of Variant No. 1.

The most important feature of puckered stripes is their wavy shape. Due to this fact, the waviness is the topography component which characterizes the seersucker effect in the best way. 

In the majority of cases, it was stated that the fabric variant influences the waviness parameters in a statistically significant way at the 0.05 significance level. Detailed results of the one–way ANOVA and post hoc–Tukey test are presented in the Supplementary Material.

**Figure 6:**
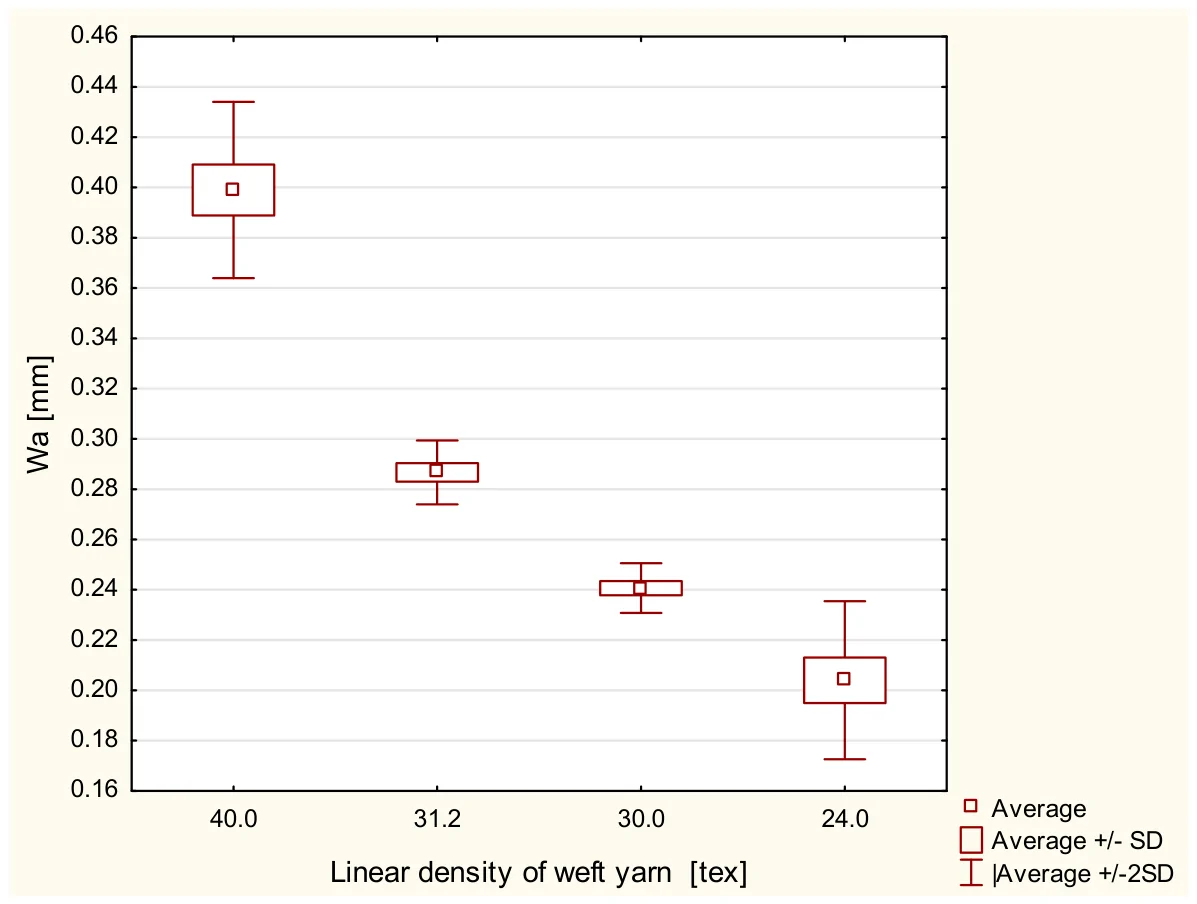
Wa parameter for the puckered stripes of the investigated seersucker woven fabrics.

Figure 6 presents the Wa parameter. It represents the arithmetic mean of the height of individual points within the evaluation area. Wa is a basic parameter characterizing the waviness of puckered stripes. The highest value of Wa (0.399 mm) was found for Variant No. 1 with the thickest weft yarn (20 tex x2), and the lowest (0.204 mm) for Variant No. 4 with the thinnest weft yarn (12 tex × 2). It is clearly seen that the thinner the weft yarn is, the lower the value of the Wa parameter (average waviness).

**Figure 7:**
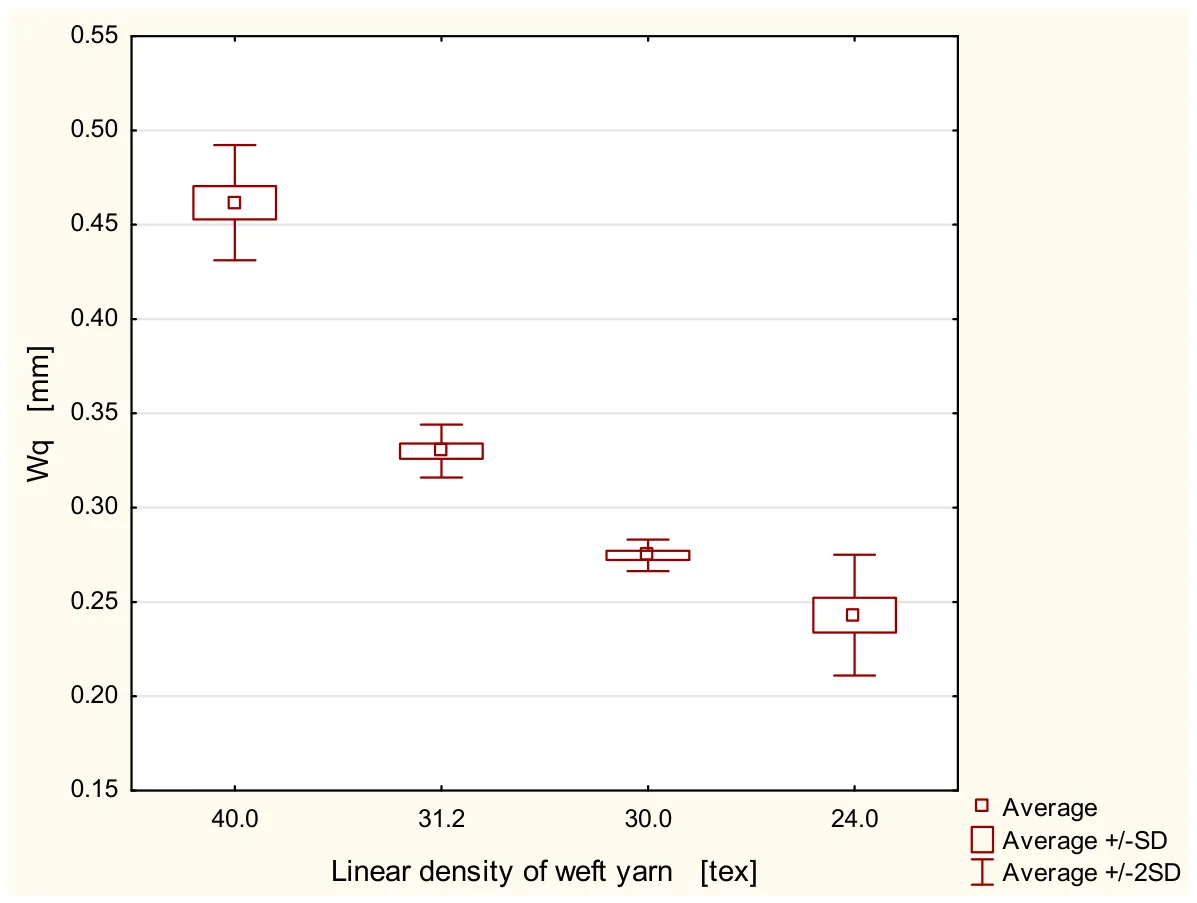
The Wq parameter for the puckered stripes of the investigated seersucker woven fabrics.

In the case of the Wq parameter, the relation is similar (Figure 7). Wq is defined as the root mean square deviation of points’ height within the evaluated area. The highest value of Wq (0.462 mm) was found for Variant No. 1 with the thickest weft yarn (20 tex × 2), while the lowest Wq (0.243 mm) occurred for Variant No. 4 with the thinnest weft yarn (12 tex × 2). Here, a straight relationship between the linear density of the yarn and the value of the Wq parameter is clearly visible. The greater linear density of the weft yarn, the higher the Wq value. 

For both parameters, Wa and Wq, Tukey’s test showed that statistically significant differences occurred between all variants (Table S2 in the Supplementary Material).

**Figure 8:**
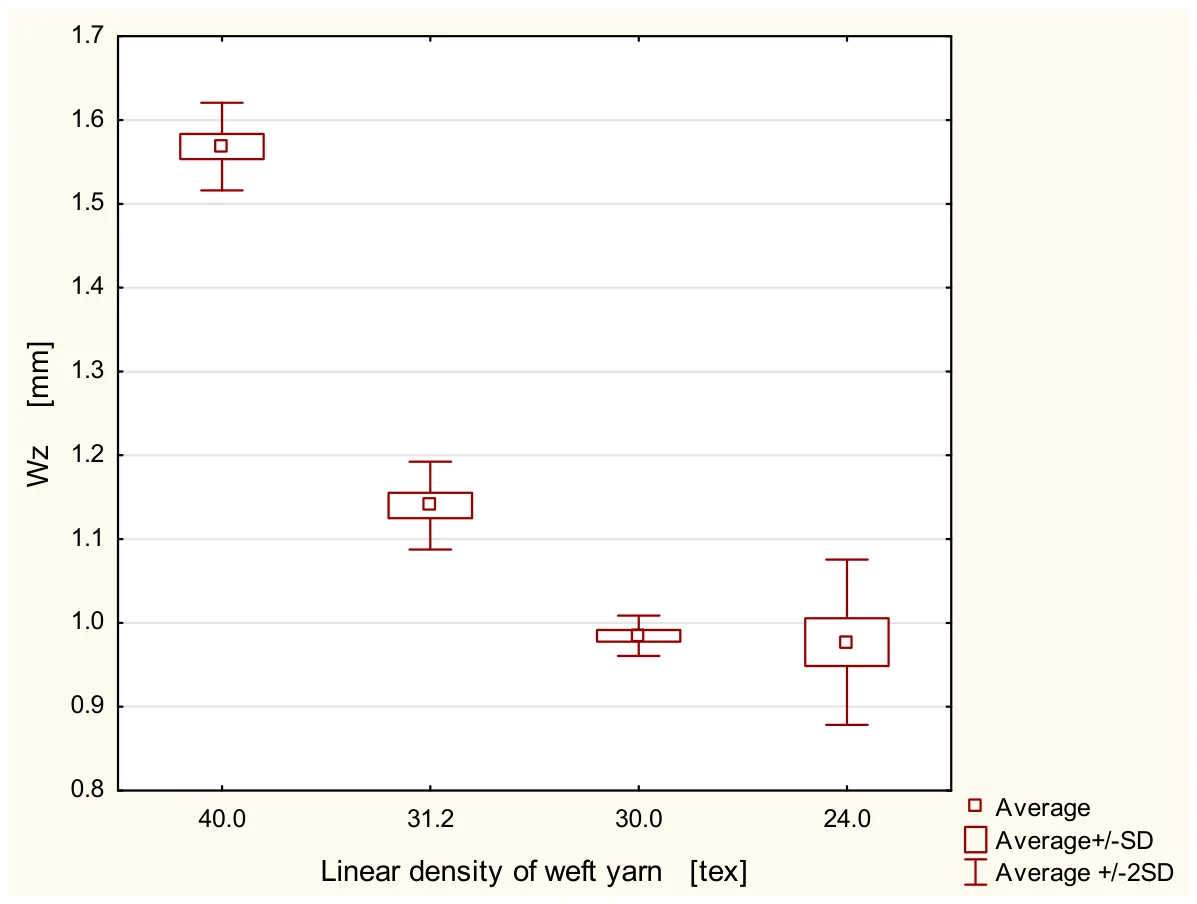
The Wz parameter for the puckered stripes of the investigated seersucker woven fabrics.

The same relation was stated for Wz (Figure 8) and other amplitude parameters: Wp, Wv and Wt. Wz is the maximum waviness height of a surface, and it is the sum of Wp (maximum peak height, which indicates the point along the sampling length at which the curve is highest) and Wv (maximum valley depth). Wt is the total height of a profile, measured as the vertical distance between the maximum profile peak height and the maximum profile valley depth across the evaluation length. It should be mentioned here that the evaluation length must contain at least one sampling length. In the case of the Wz parameter, the Tukey test showed that the difference between Variants No. 3 and No 4 is statistically insignificant (Table S2 in Supplementary Material).

**Figure 9:**
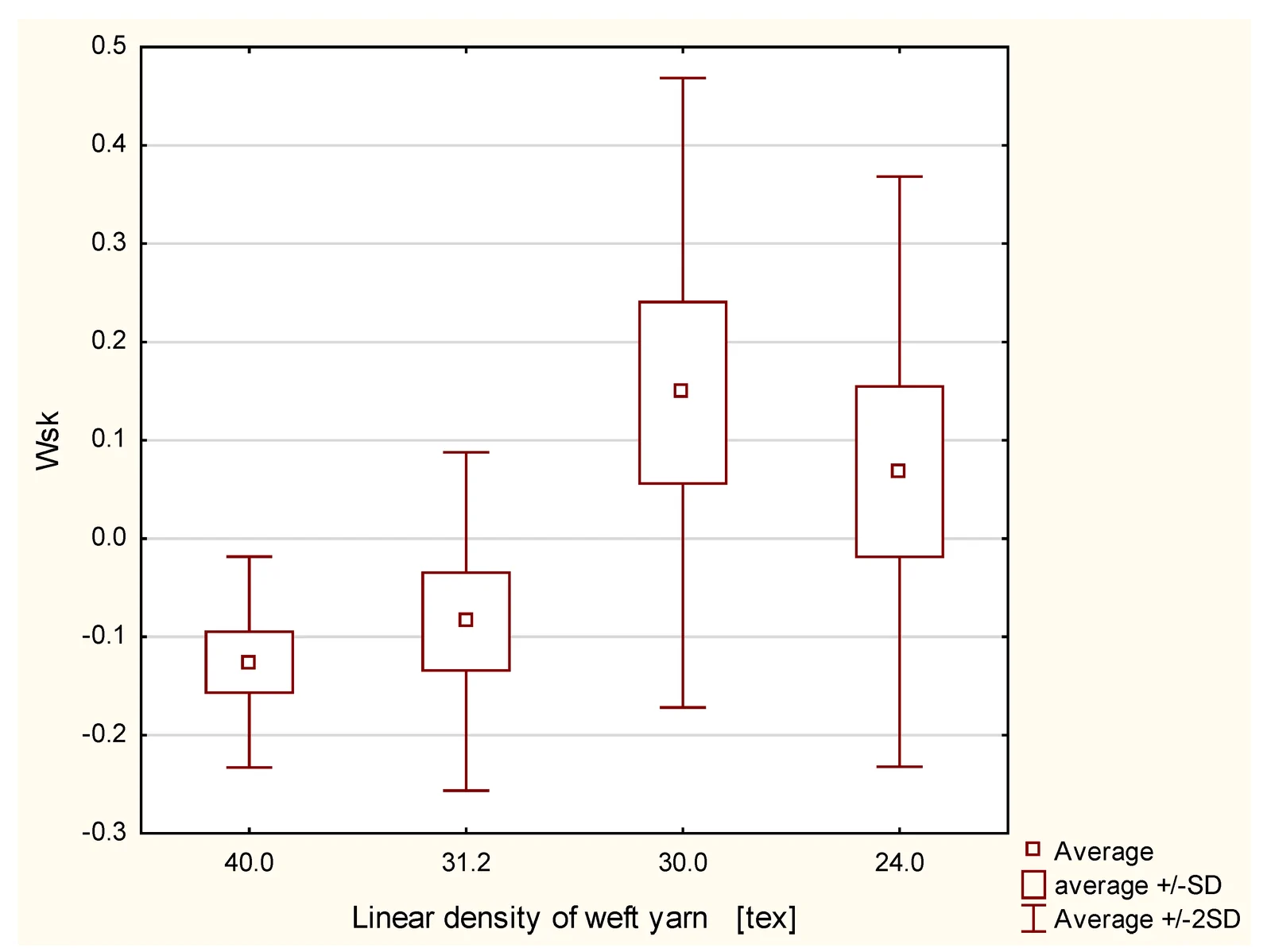
The Wsk parameter for the puckered stripes of the investigated seersucker woven fabrics.

Different relationships were found for the Wsk and Wku parameters. These parameters are of a different nature than the previously discussed Wa, Wq, and Wz. The Wsk (skewness) parameter characterizes the height distribution, particularly its shape. When Wsk is equal to zero, it means that the height distribution is symmetric relative to the mean line. In other words, it means a normal height distribution. A Wsk value higher than zero (Wsk > 0) means a deviation of the height distribution beneath the mean line. Otherwise (Wsk < 0), we are dealing with a deviation above the mean line. In the case of the investigated fabrics, the Wsk mean value for Variants No. 3 and 4 is greater than 0, 0.1483 for Variant No. 3 and 0.068 for Variant No. 4 (Figure 9). This means that the character of the height distribution in the puckered stripes of these fabrics deviates beneath the mean line. However, it should be noted that for Variant No. 4, the Wsk value is minimally greater than zero. It can be assumed that for this fabric, the distribution of point heights on the surface is very close to normal. For the Variants No.1 and No.2, the Wsk values are slightly lower than zero, −0.1257 for Variant No. 1 and −0.843 for Variant No. 2. This shows the deviation above the mean line. Statistical analysis showed that the differences between the values of Wsk for particular fabric variants are statistically insignificant at the 0.05 significance level (Table S1 in Supplementary Material). This is due to the high intra–group variability of the Wsk parameter value. 

The Wku parameter is called kurtosis. It is a statistical measure used in surface metrology to describe the distribution of peaks and valleys along the waviness profile. The interpretation of the Wku value is as follows:

Wku = 3 means a normal distribution,

Wku > 3 shows that the height distribution is sharp,

Wku < 3 means an even distribution of height.

**Figure 10:**
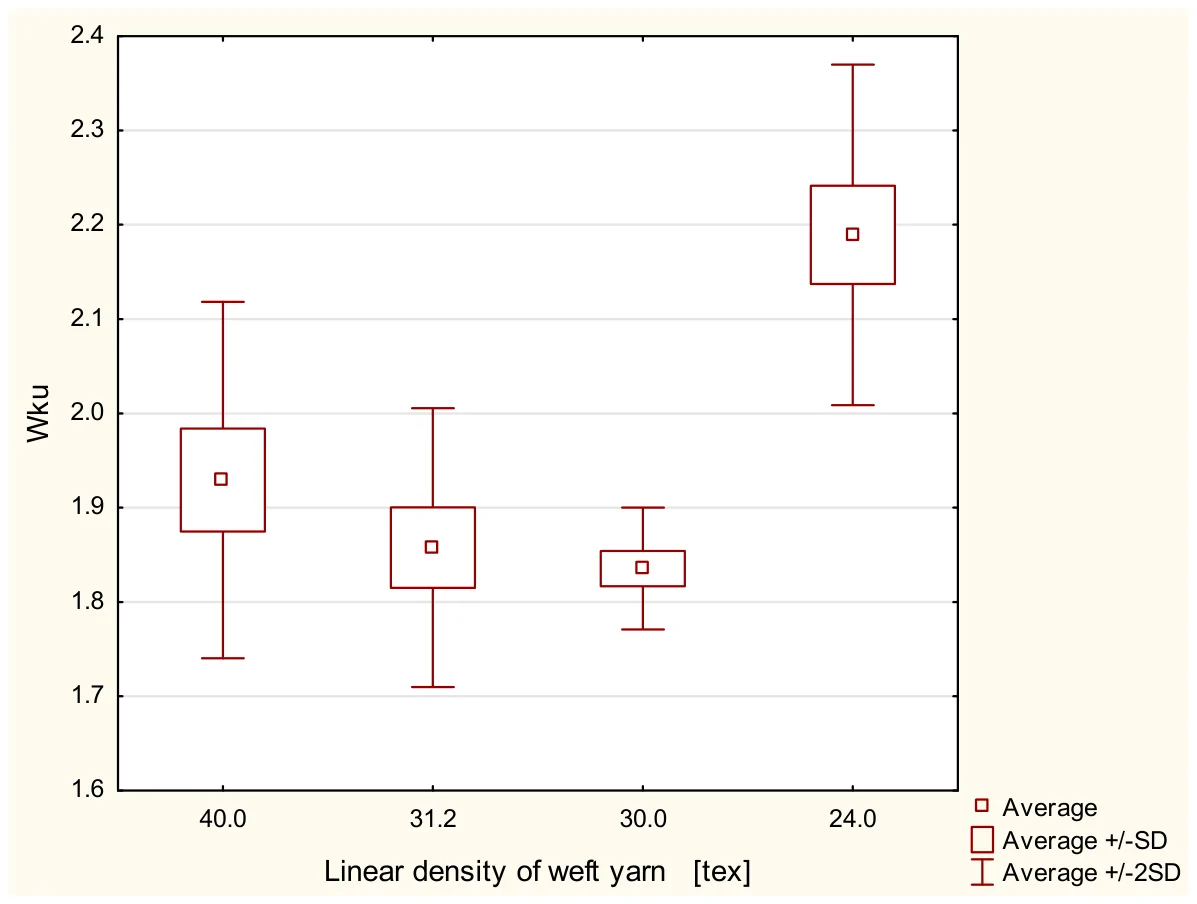
The Wku parameter for the puckered stripes of the investigated seersucker woven fabrics.

In all cases, the values of Wku for the investigated seersucker woven fabrics are lower than three (Figure 10). This shows that the distribution of peaks and valleys in the puckered stripes has the same even character for each fabric variant. ANOVA confirmed that the influence of the kind of weft yarn on kurtosis is statistically significant (Table S1 in Supplementary Material). The Tukey test showed that a statistically significant difference occurred between Variant No. 2 and the rest of the investigated fabrics (Table S2 in Supplementary Material).

Standards [26,27] describe a number of other parameters characterizing surface waviness. In addition to amplitude parameters and average amplitude parameters, these include spacing parameters, hybrid parameters, and parameters derived from the material curve. In the current work, only amplitude parameters have been analyzed and discussed. These parameters are most commonly measured and analyzed in surface studies. It should be mentioned that the parameters characterizing the waviness of the puckered stripes in seersucker woven fabrics are difficult to interpret. No such studies have been conducted to date, so there is no reference to support the analysis. Due to this fact, only the basic parameters are presented and analyzed, although all standardized parameters were determined using the described instrument and software. 

One thing can be said with certainty: the type of weft yarn used has a statistically significant effect on the values of parameters characterizing the waviness of the puckered stripes. In most cases, the value of the waviness parameter is not correlated with the linear density of the weft yarn. But for the discussed parameters, Wa, Wq, Wz, Wv and Wt, the values of the correlation coefficient are greater than 0.9 and the relationship is statistically significant. The values of correlation parameters are as follows:

- For Wa—0.97,
- For Wq—0.96,
- For Wz—0.93,
- For Wv—0.92,
- For Wt—0.92.

The number of observations is small. Making inferences about the entire population would require a larger number of observations. 

A correlation analysis was also conducted between the values of parameters characterizing the waviness of the puckered stripes and the puckering warp take–up. The analysis showed that the values of the correlation coefficients are lower than those in the case of the weft linear density and are, respectively:

- For Wa—0.72,
- For Wq—0.75,
- For Wz—0.84,
- For Wv—0.83,
- For Wt—0.86.

**Figure 11:**
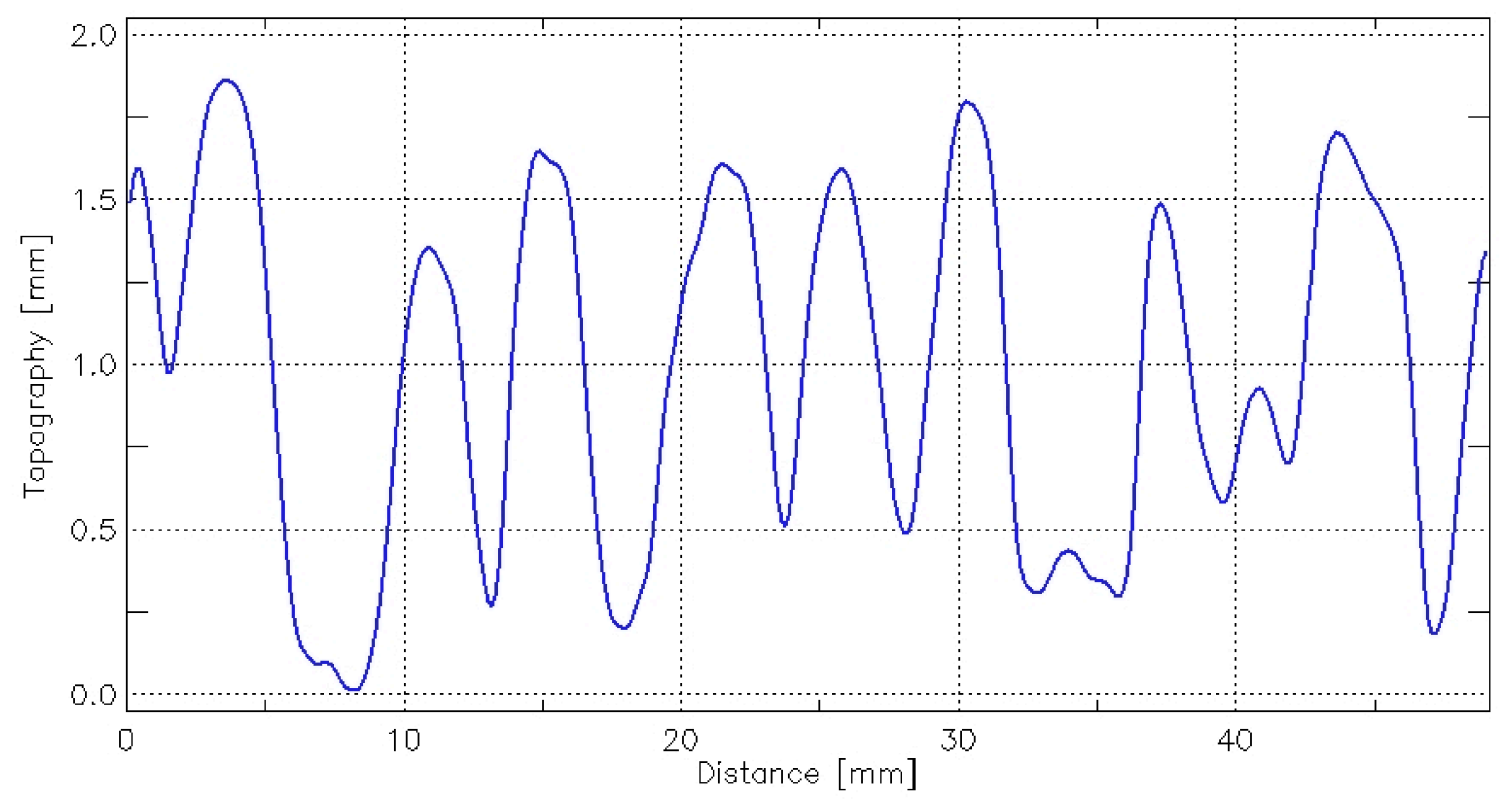
Example profile along the puckered stripe of Variant No. 1.

**Figure 12:**
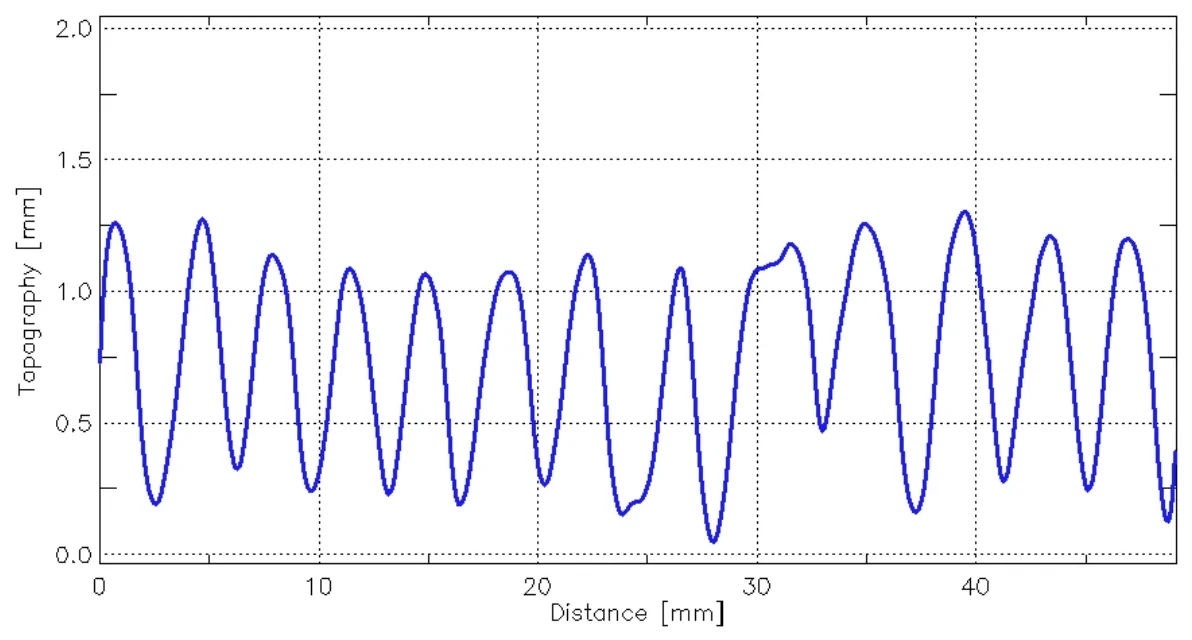
Example profile along the puckered stripe of Variant No. 2.

**Figure 13:**
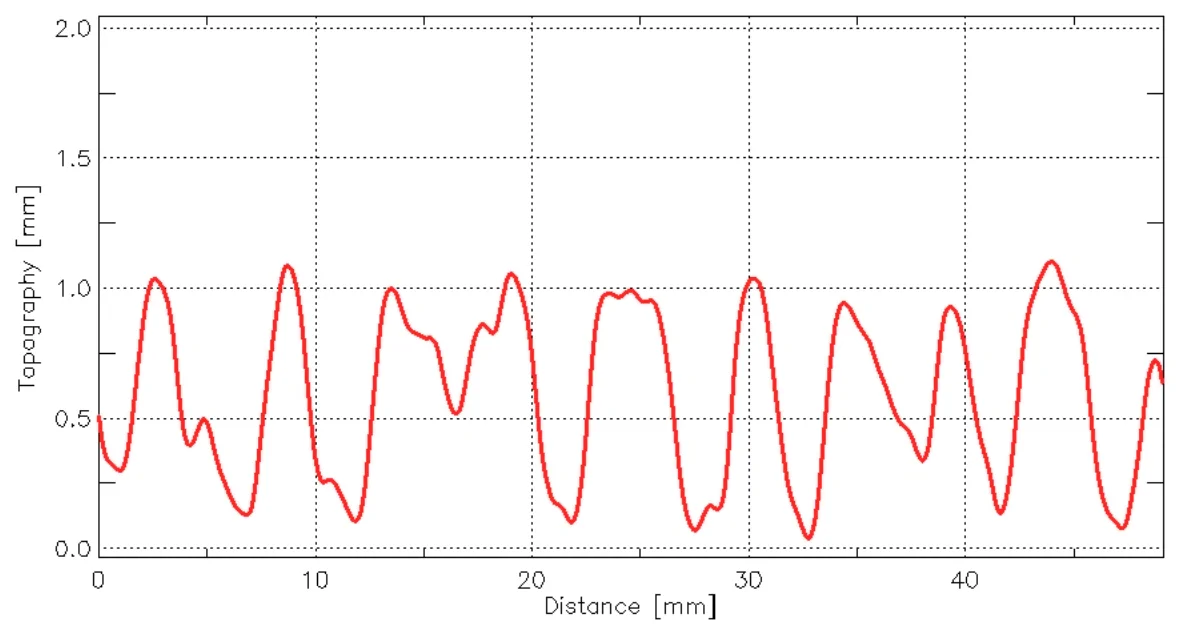
Example profile along the puckered stripe of Variant No. 3.

**Figure 14:**
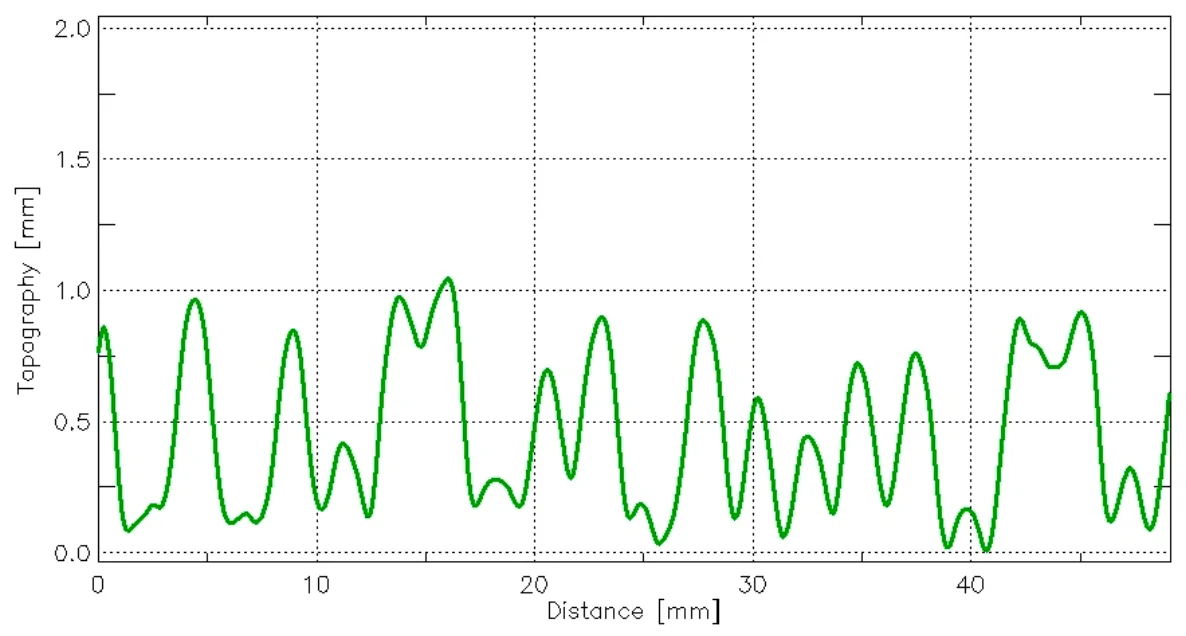
Example profile along the puckered stripe of Variant No. 4.

To assess the shape of the puckered stripes in the tested fabrics, profiles of the fabrics were generated along these stripes. The profiles were created in the center of each puckered stripe being analyzed. Example profile shapes are presented in the graphs (Figure 11, Figure 12, Figure 13 and Figure 14). 

The performed analysis confirmed that the shape of the profiles generated along the puckered stripes differs depending on the seersucker fabric variant. The differences concern both the height of the peaks on the profiles and the wave frequency, i.e., the distance between successive peaks. The highest peaks were recorded along the puckered stripes of Variant No. 1, at greater than 1.5 mm, and the lowest were recorded for Variant No. 4, mostly lower than 1 mm. These correspond to the values of Wz—the maximum waviness height of a surface (Figure 8). Additionally, in the case of Variant No. 4, small depressions were observed in the highest parts of the profile, as well as irregularly distributed peaks and valleys. In the remaining variants, especially Variants No. 1 and No. 2, the wavy profile line is very regular (Figure 11 and Figure 12).

It is also clearly seen that depending on the fabric variant, the number of peaks on the analyzed length of the puckered stripe varies. The highest number of peaks (13) occurs along the puckered stripe of Variant No. 2 (Figure 12), while the fewest peaks (9) occur in the puckered stripe of Variant No. 1 (Figure 11). Taking into account the analyzed length of the puckered stripe of 49 mm, the average distance between the consecutive peaks in Variant No. 2 is 3.8 mm, while in Variant No. 1 it is 5.4 mm. But the reported values are illustrative rather than representative characteristics of each fabric variant. There are parameters and functions such as Wsm, autocorrelation and power spectral density functions, which allow for assessment of the periodicity of waviness. This will be the topic of further investigations. 

**Figure 15:**
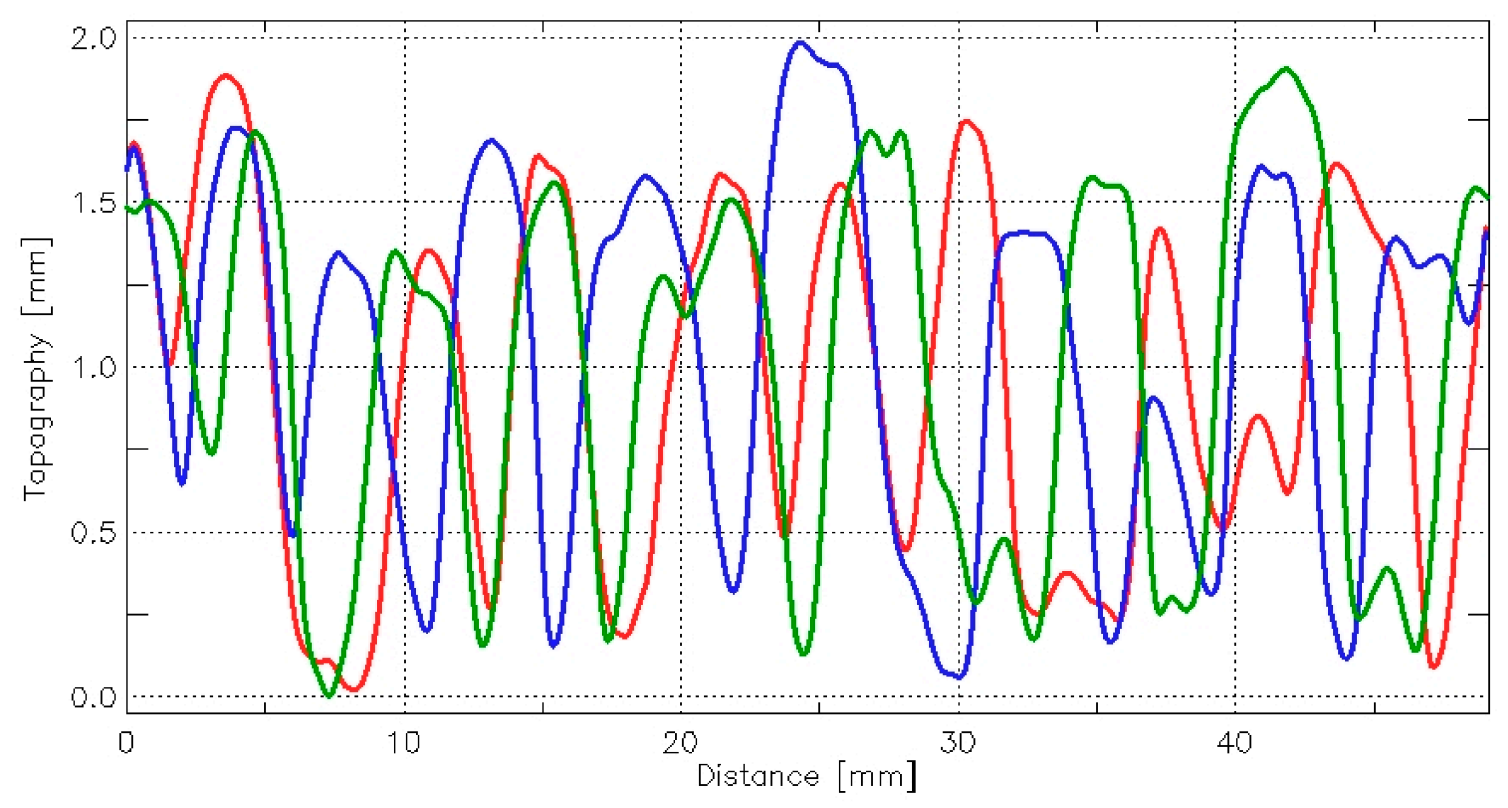
Example profiles along three adjacent puckered stripes of Variant No. 1: red line—the profile of center puckered strip; green and blue lines—profiles of puckered stripes neighboring to central puckered strip.

**Figure 16:**
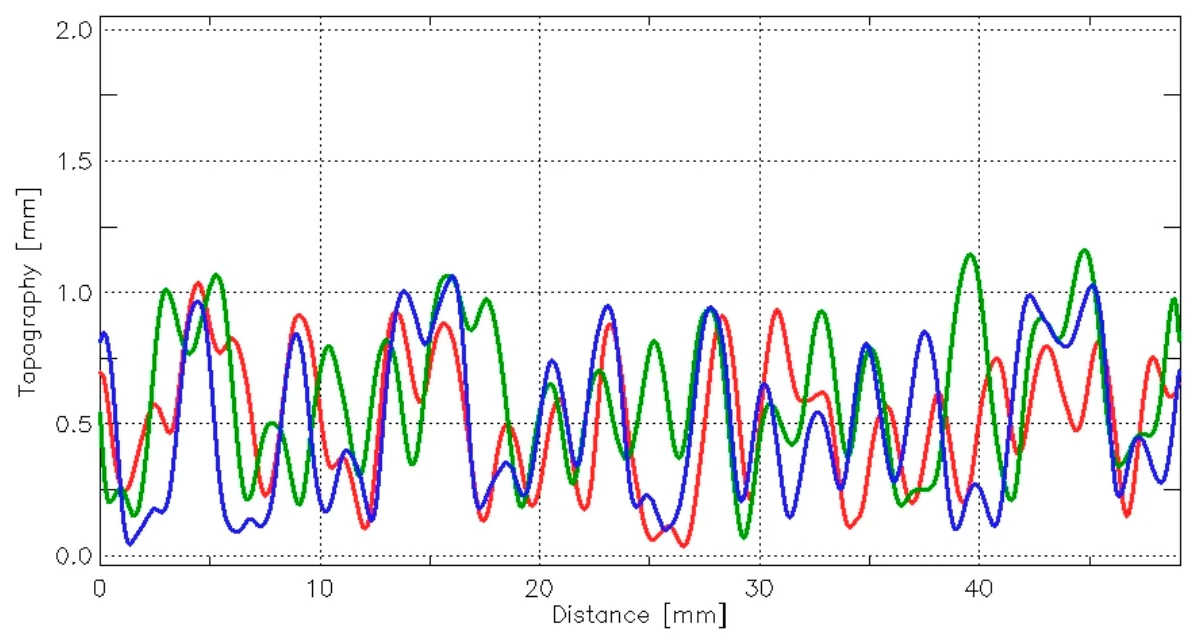
Example profiles along three adjacent puckered stripes of Variant No. 4: blue line—the profile of center puckered strip: green and red lines—profiles of puckered stripes neighboring to central puckered strip.

Figure 15 and Figure 16 present example profiles along three adjacent puckered stripes of Variants No. 1 and No. 4. 

It is clearly visible that the profile shapes of neighboring puckered stripes are very similar. There is a shift in the peaks and valleys along the stripes’ length. Similar observations were also made for Variants No. 2 and No. 3. This shows that the shape of the profiles of puckered stripes can be a useful tool in assessing the repeatability and reproducibility of the seersucker effect in subsequent batches of seersucker woven fabrics.

The Mark III software provides many more possibilities to analyze surface topography. It is possible to create histograms presenting the distribution of points’ height on the measured surface, the autocorrelation function, angle distribution, power spectra density, material ratio curve and others. Some tools are not available for examining only selected sections of the image obtained from a profilometer. The lack of scientific publications on this topic hinders the use of these tools. Research will be continued. The goal will be to attempt to quantify the surface geometry of puckered stripes in seersucker fabrics. This will allow for numerical determination of the shape of the puckered phase in these fabrics, thus enabling verification of the repeatability and reproducibility of these shapes.

## 4. Conclusions

Research has shown that a non–contact surface measurement method enables an assessment of the surface geometry of the puckered phase of seersucker woven fabrics. The optical profilometer provides a range of parameters, functions, and other tools characterizing the surface geometry of puckered stripes.

Studies of seersucker woven fabrics made from the same warp yarns, differing in the kind of weft yarn used, showed that the use of a specific weft yarn allows for properly shaping the puckered area of the fabrics. The results showed that the kind of weft yarn influences the waviness amplitude parameters and shape of the puckered stripes in a statistically significant way. It was observed that weft yarns with higher linear density provide the most intense seersucker effect, meaning a wave with the greatest peak height and greatest valley depth. Conversely, weft yarns with the lowest linear density provide less variation in peak height and valley depth but higher wave frequency. However, it should be mentioned here that the observed differences are associated with the weft yarn type, with linear density being only one of several possible explanatory factors.

The research requires continuation in order to check the usefulness of other tools available in the 3D contactless measurement of surfaces for assessing and quantifying the shape of the puckered surfaces of seersucker woven fabrics.

## Figures and Tables

**Table 1:** The basic structural parameters of the investigated seersucker woven fabrics.

Parameter	Unit	Value			
		Variant 1	Variant 2	Variant3	Variant 4
Raw material—warp I	–	CO	CO	CO	CO
Raw material—warp II	–	CO	CO	CO	CO
Raw material—weft	–	CO	ThermoCool Fresh 156/94/2	CO50/PES50	Dry Release See Cell active
Warp I linear density	tex	20 tex × 2	20 tex × 2	20 tex × 2	20 tex × 2
Warp II linear density	tex	20 tex × 2	20 tex × 2	20 tex × 2	20 tex × 2
Weft linear density	tex	20 × 2	15.6 × 2	15 × 2	12.0 × 2
Weave—warp I	–	plain	plain	plain	plain
Weave—warp II	–	rep 2/2	rep 2/2	rep 2/2	rep 2/2
Warp density	cm^−1^	27.8	28.4	28.1	27.4
Weft density	cm^−1^	22.1	29.0	29.0	28.1
Mass per square meter	gm^−2^	241	201	224	208
Warp I take–up	%	5.12	1.85	3.32	5.06
Warp II take–up	%	70.6	60.0	54.5	62.9
Weft take–up	%	13.7	15.9	13.0	10.3

## Data Availability

The data presented in this study are available on request from the corresponding author.

## Supplementary Materials

The following supporting information can be downloaded at: https://www.mdpi.com/article/10.3390/ma19173648/s1, Table S1. One-way ANOVA results for waviness parameters; Table S2. Tukey’s test results.
