# Supplementary material for: Assessment of Surface Geometry of Puckered Area of SeerSucker Woven Fabrics
Source: Materials (Basel). 2026 Aug 27;19(17):3648. doi: 10.3390/ma19173648 (PMC13566725; doi:10.3390/ma19173648)
Supplement: Supplementary file 1 [file materials-19-03648-s001.zip › materials-4496875-supplementary.pdf]

Table S1. One-way ANOVA results for waviness parameters

Parameter	One-way ANOVA results							
	SS Effect	df Efect	MS Efect	SS Error	df Error	MS Error	F	p
Wa	0,064506	3	0,021502	0,001237	8	0,000155	139,0207	0,000000
Wq	0,083815	3	0,027938	0,001111	8	0,000139	201,1166	0,000000
Wz	0,693426	3	0,231142	0,007889	8	0,000986	234,3842	0,000000
Wp	0,261162	3	0,087054	0,007976	8	0,000997	87,31566	0,000002
Wv	0,357066	3	0,119022	0,006705	8	0,000838	142,0027	0,000000
Wsk	0,0130	4	0,00324	0,14021	20	0,007011	0,4620	0,762731
Wku	0,238042	3	0,079347	0,047179	8	0,005897	13,45480	0,001716

Legend: SS – sum of squares, df – degree of freedom, MS – mean square, F – variable of F distribution, p – statistical significance.

Table S2. Tukey's test results

Weft yarn	20 tex x 2	15.6 tex x 2	15 tex x 2	12 tex x 2
		Wa		
20 tex x 2		0,000236	0,000231	0,000231
15.6 tex x 2	0,000236		0,008443	0,000369
15 tex x 2	0,000231	0,008443		0,028391
12 tex x 2	0,000231	0,000369	0,028391	
		Wq		
20 tex x 2		0,000231	0,000231	0,000231
15.6 tex x 2	0,000231		0,002072	0,000282
15 tex x 2	0,000231	0,002072		0,044280
12 tex x 2	0,000231	0,000282	0,044280	
		Wz		
20 tex x 2		0,000231	0,000231	0,000231
15.6 tex x 2	0,000231		0,001524	0,001155
15 tex x 2	0,000231	0,001524		0,990063
12 tex x 2	0,000231	0,001155	0,990063	
		Wp		
20 tex x 2		0,000233	0,000231	0,000231
15.6 tex x 2	0,000233		0,330338	0,396614
15 tex x 2	0,000231	0,330338		0,998671
12 tex x 2	0,000231	0,396614	0,998671	
		Wv		
20 tex x 2		0,000233	0,000231	0,000231
15.6 tex x 2	0,000233		0,001347	0,001438
15 tex x 2	0,000231	0,001347		0,999872
12 tex x 2	0,000231	0,001438	0,999872	
		Wsk		
20 tex x 2		0,973732	0,091230	0,277179
15.6 tex x 2	0,973732		0,163681	0,457860
15 tex x 2	0,091230	0,163681		0,846343
12 tex x 2	0,277179	0,457860	0,846343	
		Wku		
20 tex x 2		0,675649	0,480645	0,013819
15.6 tex x 2	0,675649		0,983468	0,003404
15 tex x 2	0,480645	0,983468		0,002313
12 tex x 2	0,013819	0,003404	0,002313	
